# Synthesis and evaluation of a novel PET ligand, a GSK’963 analog, aiming at autoradiography and imaging of the receptor interacting protein kinase 1 in the brain

**DOI:** 10.1186/s41181-023-00217-z

**Published:** 2023-10-18

**Authors:** Hiroshi Ikenuma, Aya Ogata, Hiroko Koyama, Bin Ji, Hideki Ishii, Takashi Yamada, Junichiro Abe, Chie Seki, Yuji Nagai, Masanori Ichise, Takafumi Minamimoto, Makoto Higuchi, Ming-Rong Zhang, Takashi Kato, Kengo Ito, Masaaki Suzuki, Yasuyuki Kimura

**Affiliations:** 1 Department of Clinical and Experimental Neuroimaging, Center for Development of Advanced Medicine for Dementia, National Center for Geriatrics and Gerontology (NCGG), 7-430 Morioka-Cho, Obu, Aichi 474-8511 Japan; 2 Department of Pharmacy, Faculty of Pharmacy, Gifu University of Medical Science (GUMS), Kani, Japan; 3 Department of Chemistry and Biomolecular Science, Faculty of Engineering, Gifu University, Gifu, Japan; 4 Department of Functional Brain Imaging, National Institutes for Quantum Science and Technology (QST), Chiba, Japan; 5 Department of Radiopharmacy and Molecular Imaging, School of Pharmacy, Fudan University, Shanghai, China; 6 Department of Advanced Nuclear Medicine Sciences, National Institutes for Quantum Science and Technology (QST), Chiba, Japan

**Keywords:** Receptor interacting protein kinase 1, Alzheimer’s disease, Positron emission tomography

## Abstract

**Background:**

Receptor interacting protein kinase 1 (RIPK1) is a serine/threonine kinase, which regulates programmed cell death and inflammation. Recently, the involvement of RIPK1 in the pathophysiology of Alzheimer’s disease (AD) has been reported; RIPK1 is involved in microglia’s phenotypic transition to their dysfunctional states, and it is highly expressed in the neurons and microglia in the postmortem brains in AD patients. They prompt neurodegeneration leading to accumulations of pathological proteins in AD. Therefore, regulation of RIPK1 could be a potential therapeutic target for the treatment of AD, and in vivo imaging of RIPK1 may become a useful modality in studies of drug discovery and pathophysiology of AD. The purpose of this study was to develop a suitable radioligand for positron emission tomography (PET) imaging of RIPK1.

**Results:**

(*S*)-2,2-dimethyl-1-(5-phenyl-4,5-dihydro-1*H*-pyrazol-1-yl)propan-1-one (GSK’963) has a high affinity, selectivity for RIPK1, and favorable physiochemical properties based on its chemical structure. In this study, since ^11^C-labeling (half-life: 20.4 min) GSK’963 retaining its structure requiring the Grignard reaction of *tert*-butylmagnesium halides and [^11^C]carbon dioxide was anticipated to give a low yield, we decided instead to ^11^C-label a GSK’963 analog ((*S*)-2,2-dimethyl-1-(5-(*m*-tolyl)-4,5-dihydro-1*H*-pyrazol-1-yl)propan-1-one, GG502), which has a high RIPK1 inhibitory activity equivalent to that of the original compound GSK’963. Thus, we successfully ^11^C-labeled GG502 using a Pd-mediated cross-coupling reaction in favorable yields (3.6 ± 1.9%) and radiochemical purities (> 96%), and molar activity (47–115 GBq/μmol). On autoradiography, radioactivity accumulation was observed for [^11^C]GG502 and decreased by non-radioactive GG502 in the mouse spleen and human brain, indicating the possibility of specific binding of this ligand to RIPK1. On brain PET imaging in a rhesus monkey, [^11^C]GG502 showed a good brain permeability (peak standardized uptake value (SUV) ~3.0), although there was no clear evidence of specific binding of [^11^C]GG502. On brain PET imaging in acute inflammation model rats, [^11^C]GG502 also showed a good brain permeability, and no significant increased uptake was observed in the lipopolysaccharide-treated side of striatum. On metabolite analysis in rats at 30 min after administration of [^11^C]GG502, ~55% and ~10% of radioactivity was from unmetabolized [^11^C]GG502 in the brain and the plasma, respectively.

**Conclusions:**

We synthesized and evaluated a ^11^C-labeled PET ligand based on the methylated analog of GSK’963 for imaging of RIPK1 in the brain. Although in autoradiography of the resulting [^11^C]GG502 indicated the possibility of specific binding, the actual PET imaging failed to detect any evidence of specific binding to RIPK1 despite its good brain permeability. Further development of radioligands with a higher binding affinity for RIPK1 in vivo and more stable metabolite profiles compared with the current compound may be required.

**Supplementary Information:**

The online version contains supplementary material available at 10.1186/s41181-023-00217-z.

## Introduction

Receptor interacting protein kinase 1 (RIPK1) is a serine/threonine kinase, which regulates programmed cell deaths, where necroptosis and apoptosis are initiated by the tumor necrosis factor receptor 1 and tumor necrosis factor-α in inflammatory responses (Bell et al. 2008; Stanger et al. 1995). Activation of RIPK1 increases the release of damage-associated molecular patterns from the necrotic cells; promotes the inflammatory gene expression in microglia; and increases the release of inflammation-inducing cytokines from macrophages, resulting in active inflammation reactions (Degterev et al. 2019).

Recently, involvement of RIPK1 in the pathophysiology of Alzheimer’s disease (AD) has been reported; RIPK1 is highly expressed in the neurons and microglia in the postmortem brains of AD patients (Ofengeim et al. 2017). RIPK1 is highly expressed during microglia’s phenotypic transition from their homeostatic states to their dysfunctional states, the latter state being characterized by the reduced capacity for phagocytosis and clearance of amyloid β proteins (Ofengeim et al. 2017). They prompt neurodegeneration to proceed, leading to the accumulation of the pathological proteins in AD. Therefore, regulation of RIPK1 could be a potential therapeutic target for the treatment of AD, and in vivo imaging of RIPK1 may become a useful modality in studies of drug discovery and pathophysiology of AD.

**Fig. 1:**
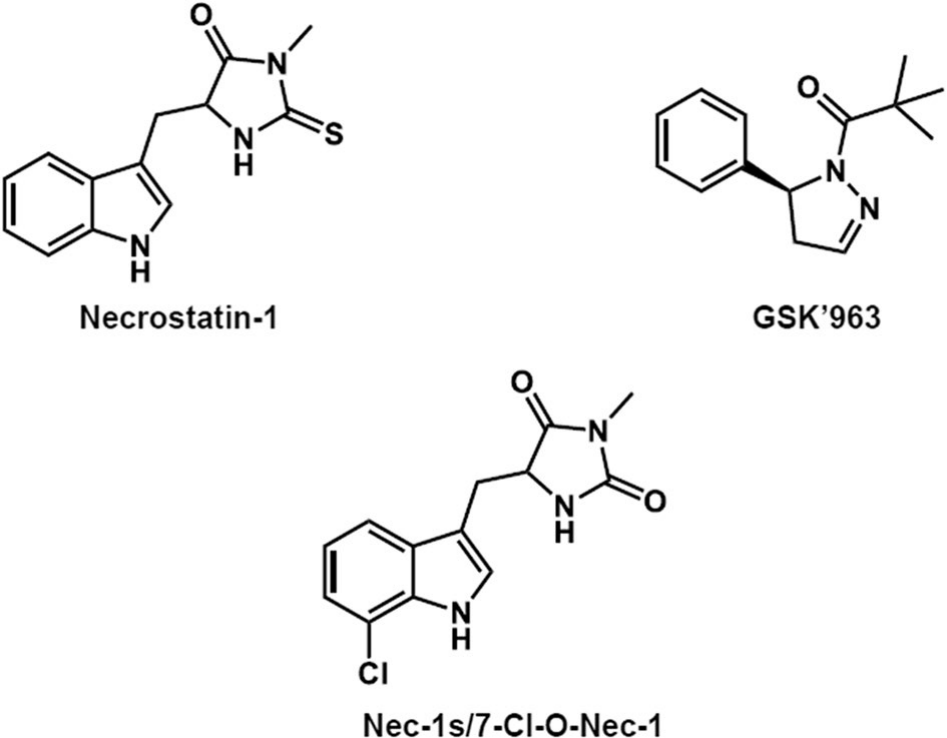
Receptor-interacting protein kinase 1 (RIPK1) inhibitors

To this end, several small molecules with RIPK1 inhibitory activity have been reported by other investigators (Fig. 1). Degterev et al. reported that 5-(indol-3-ylmethyl)-(2-thio-3-methyl)hydantoin (Necrostatin-1) targets RIPK1, showing its cytoprotection against tumor necrosis factor-α-induced necroptosis in Fas-associated protein with death domain-deficient jurkat cells at 50% effective concentration (*EC*_50_) = 494 nM (Degterev et al. 2005). However, Necrostatin-1 has a non-selective inhibitory activity against indoleamine-2,3-dioxigenase, an immune regulating oxidoreductase (Takahashi et al. 2012). On the other hand, Takahashi et al. developed 5-((7-chloro-1*H*-indol-3-yl)methyl)-3-methyl-2,4-imidazolidinedione (Nec-1s or 7-Cl-O-Nec-1), a derivative of Necrostatin-1, which has RIPK1 inhibitory activity equivalent to that of Necrostatin-1 without posing any off-target activity against indoleamine-2,3-dioxigenase unlike Necrostatin-1 (Takahashi et al. 2012). Nec-1s has a > 1000 times higher selectivity to RIPK1 than other human kinases (Takahashi et al. 2012). Recently, Berger et al. also developed a novel RIPK1 inhibitor, (*S*)-2,2-dimethyl-1-(5-phenyl-4,5-dihydro-1*H*-pyrazol-1-yl)propan-1-one (GSK’963), that has higher selectivity and > 200 times higher RIPK1inhibitory activity than does Necrostatin-1 (Berger et al. 2015).

**Fig. 2:**
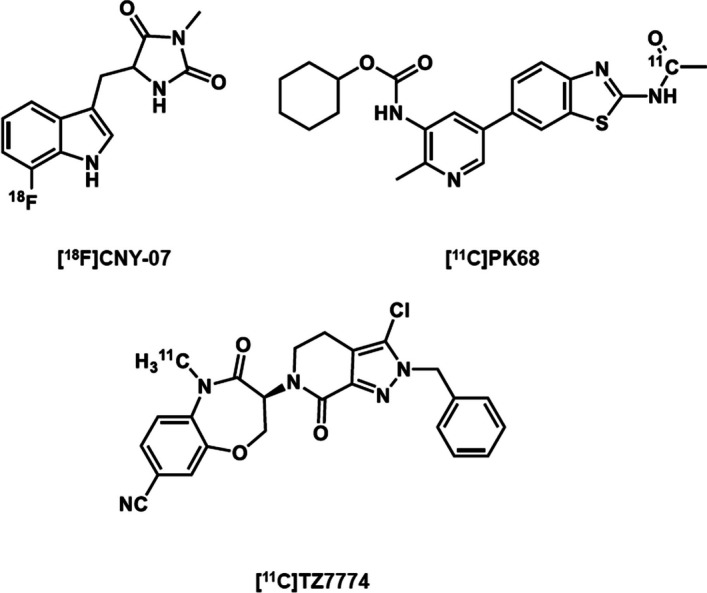
PET ligands targeting receptor-interacting protein kinase 1 (RIPK1)

Based on the chemical structures of these RIPK1 inhibitors, three ligands for positron emission tomography (PET) imaging of RIPK1 have so far been developed by others. A PET ligand [^18^F]CNY-07 (Fig. 2) based on the chemical structure of Nec-1s showed its radioactive uptake in the whole mouse brain was as much as 3% ID/cc (Lan et al. 2021). However, its radioactive uptake reflected no clear specific binding. Another PET ligand targeting RIPK1, [^11^C]PK68 (Fig. 2), likewise showed neither clear specific binding nor significant brain permeability in healthy mice (Yamasaki et al. 2022). Finally, [^11^C]TZ7774 (Fig. 2) showed a good brain permeability in mice and a macaque but no clear evidence of RIPK1 specific binding in the brain (Huang et al. 2022).

The purpose of this study was to develop a more suitable radioligand for PET imaging of RIPK1. We decided to synthesize [^11^C]methyl-labeled GSK’963, because GSK’963 has a high affinity (50% inhibitory concentration (*IC*_50_) = 29 nM in the fluorescent polarization binding assay), a high selectivity for RIPK1 (> 10,000-fold selectivity to RIPK1 over 339 other kinases), and a favorable computed lipophilicity of 2.7 (clogP) (Berger et al. 2015). Thus, this ligand should also have a high brain permeability based on its chemical structure (see also discussion). We applied a rapid Pd^0^-mediated cross-coupling reaction between [^11^C]iodomethane and an organoboronic acid ester to label GSK’963 with [^11^C]methyl group (Suzuki et al. 2018).

## Results

### Synthesis of [^11^C]GG502 for imaging of RIPK1

**Fig. 3:**
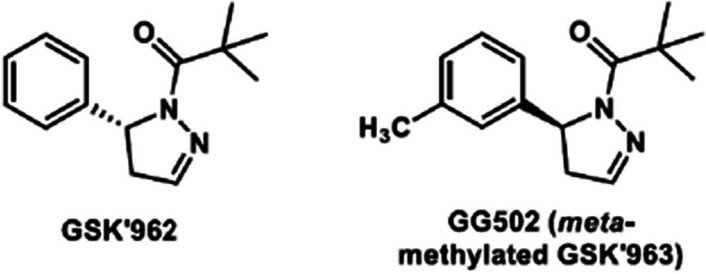
GSK’962 and design of labeled compound, GG502

The introduction of carbon-11 into the parent structure of GSK’963 using Grignard chemistry and [^11^C]carbon dioxide was expected to give a low yield. Therefore, we designed a methyl-substituted GSK’963 analog on the aromatic ring ((*S*)-2,2-dimethyl-1-(5-(*m*-tolyl) -4,5-dihydro-1*H*-pyrazol-1-yl)propan-1-one, **GG502**, Fig. 3) that could be radiolabeled using a Pd^0^-mediated cross-coupling reaction. We decided to introduce the methyl group on the *meta-*position based on the several reports showing the *para*-position on the benzene ring substituted with an electron-donating group is more susceptible to oxidative metabolism than the *meta*-position (Koyama et al. 2017; Smith et al. 1996; Thompson 2001; Van De Waterbeemd et al. 2001). GSK’962, the enantiomer of GSK’963, has a lower RIPK1 inhibitory activity, suggesting that the steric configuration near the chiral carbon atom affect RIPK1 inhibitory activity (Fig. 3). Thus, we assumed that introducing a methyl group on the *meta*-position, which is farther away from the chiral carbon atom than *ortho*-position, should maintain its RIPK1 inhibitory activity.

**Scheme 1:**
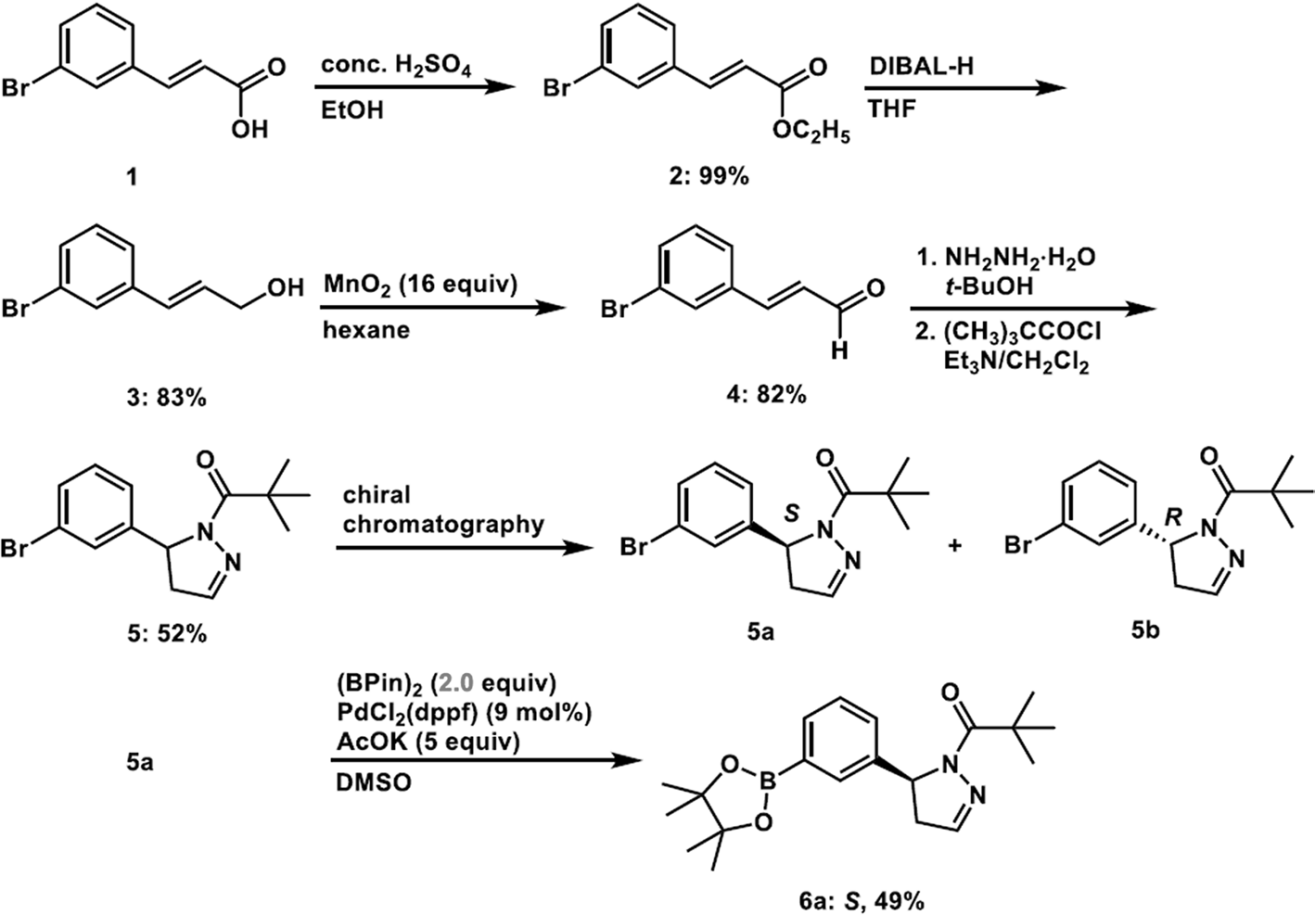
Synthesis of the boron precursor **6a.** The resulting yields of the precursor and intermediates are provided with each compound number

A pinacol borate precursor, **6a**, was synthesized via separation of the racemic bromo intermediate (**5**) between **5a** and **5b** (Scheme 1). The bromophenyl-substituted dihydropyrazole was prepared according to the reported method (Harris et al. 2019). The required aryl, *α*,*β*-unsaturated aldehyde **4** was obtained from diisobutylaluminum hydride reduction of cinnamic acid ethyl ester **2** followed by oxidation with manganese dioxide. The aryl *α*,*β*-unsaturated aldehyde **4** was cyclized to the dihydropyrazole ring **5** with hydrazine, which was subsequently acylated with an acid chloride or coupled with a carboxylic acid. The compound **5** was isolated as a racemate and submitted for chiral high-performance liquid chromatogram separation to give the active (*S*)- and (*R*)-enantiomers, **5a** and **5b**, respectively, then **5a** was converted into pinacol borate precursor **6a** using bis(pinacolato)diboron and Pd catalyst in dimethyl sulfoxide under basic conditions (see Additional file 1). The resulting yields of the precursor and intermediates are provided in the Scheme 1.

**Scheme 2:**
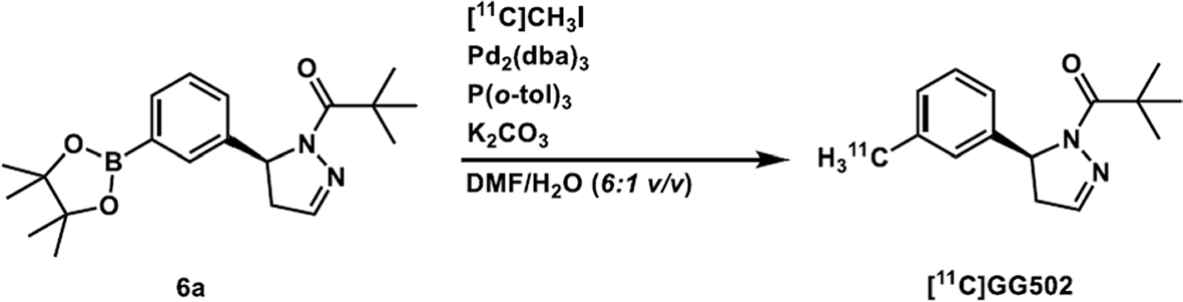
Synthesis of chiral [^11^C]methyl-labeled compound, [^11^C]GG502

Radiosynthesis of [^11^C]GG502 using rapid *C*-[^11^C]methylation of the precursor **6a** was conducted using [^11^C]iodomethane in the presence of tris(dibenzylideneacetone)dipalladium, tris(*o*-tolyl)phosphine, potassium carbonate in *N*,*N*-dimethylformamide/water (6:1 *v*/*v*) at 60 °C for 4 min (Scheme 2). Purification by preparative high performance liquid chromatography (HPLC) (see Additional file 1: Fig. S5) and radiopharmaceutical formulation gave formulated [^11^C]GG502 with high radiochemical purities (> 96%, see Additional file 1: Fig. S6). Total synthesis time taken from the end of irradiation was 33–35 min, producing radioactivities of 90–1210 MBq for [^11^C]GG502. Decay-corrected radiochemical yields of [^11^C]GG502 based on the radioactivity of [^11^C]carbon dioxide trapped in the lithium aluminum solution were 3.6 ± 1.9% (n = 14). Molar activity of [^11^C]GG502 after the formulation was in the range of 47–115 GBq/μmol (see Additional file 1: Fig. S7).

### RIPK1 kinase inhibitory assay

We evaluated whether GG502 has a high RIPK1 inhibitory activity equivalent to that of the original compound GSK’963. In the inhibitory activity assay using recombinant human RIPK1 protein, RIPK1 substrate, and adenosine triphosphate, GG502 showed *IC*_50_ of 44 nM, which was nearly the same as *IC*_50_ of GSK’963 (52 nM), thus the introduction of a methyl group on the *meta*-position of GSK’963 appeared to have a minimal effect on its high RIPK1 inhibitory property of GSK’963.

### Autoradiography

**Fig. 4:**
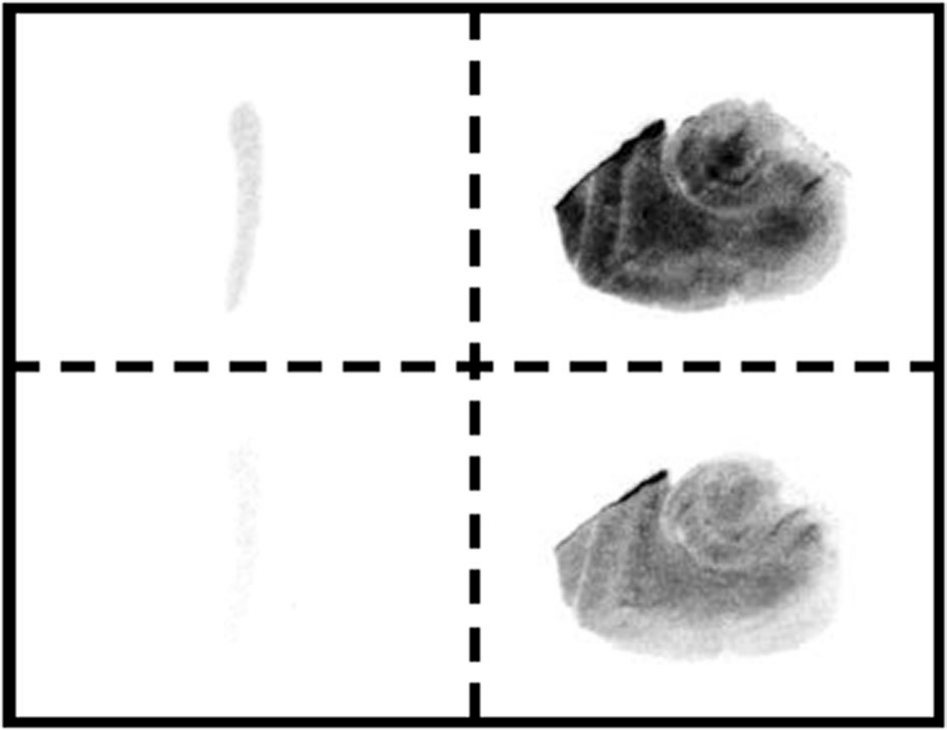
Autoradiography of [^11^C]GG502 in mouse spleen (left) and in human hippocampus of healthy subjects (right) without (top) and with addition of non-radioactive GG502 (10 μM) as a blocking agent (bottom)

To evaluate specific binding of [^11^C]GG502 to RIPK1, autoradiography was performed in the mouse spleen and human hippocampus of healthy subjects (Fig. 4). Radioactivity accumulation was observed with [^11^C]GG502 and was decreased by non-radioactive GG502 in the mouse spleen and human brain, providing evidence of specific binding of this ligand to RIPK1. In the mouse spleen sections, the radioactivity after blocking was hardly visible (Fig. 4 left bottom) when the visual scale was same as that of the human brain sections. However, when the visual scale was lowered, residual radioactivity after blocking was visible indicated that nonspecific binding of GG502 in the mouse spleen was much less compared with human brain (see Additional file 1: Fig. S8).

### Brain PET imaging in a monkey

**Fig. 5:**
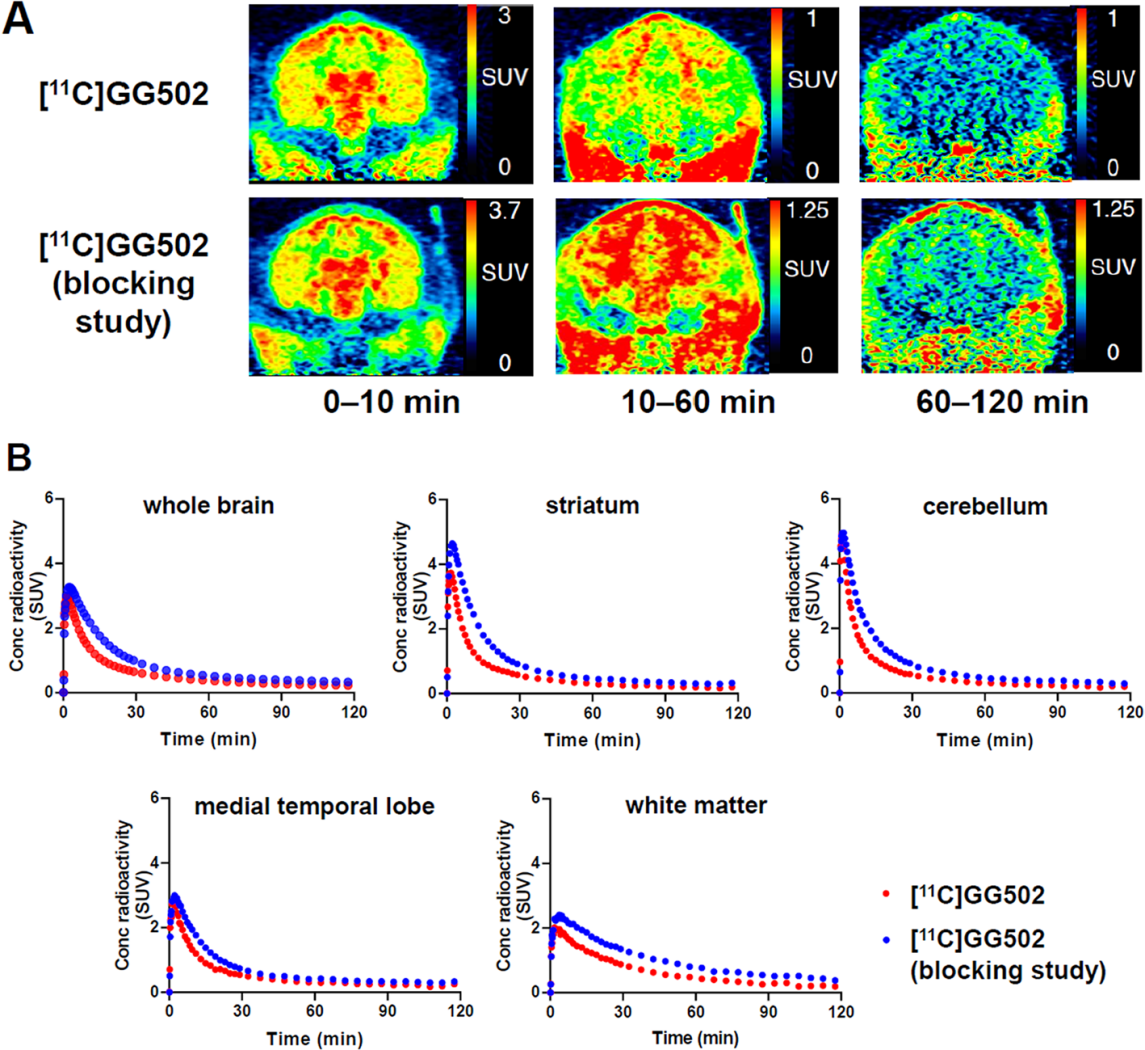
Evaluation of [^11^C]GG502 in a healthy rhesus monkey. **A** Coronal brain PET images in a rhesus monkey averaged from 0–10, 10–60, and 60–120 min after intravenous injection of [^11^C]GG502 (top), [^11^C]GG502 with pre-blocking (bottom). **B** Time activity curves of whole brain, striatum, cerebellum, medial temporal lobe, and white matter after intravenous injection of [^11^C]GG502 (red), [^11^C]GG502 with pre-blocking (blue). Non-radioactive GG502 1 mg/kg intravenous injection was performed 5 min before [^11^C]GG502 injection in a blocking study

We performed brain PET imaging with [^11^C]GG502 in a rhesus monkey. [^11^C]GG502 showed a good brain uptake (Fig. 5). To evaluate the specific radioactivity accumulation in monkey brain, we also performed brain PET imaging with pretreatment of non-radioactive GG502 intravenously injected 5 min before [^11^C]GG502 injection. After the pretreatment, radioactivity was overall increased. However, there was no clear evidence of specific binding of GG502.

### Brain PET imaging in acute inflammation model rats

**Fig. 6:**
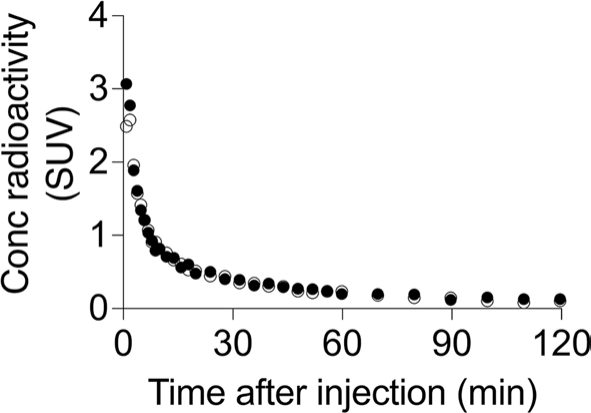
Evaluation of [^11^C]GG502 in rats with acute inflammation. Time activity curves of right (filled circle) and left (open circle) striatum in rats after intravenous injection of [^11^C]GG502. PET scans were performed 4 days after administration with 50 μg LPS on right striatum in rats

We conducted brain PET imaging after an intravenous injection of [^11^C]GG502 in acute inflammation model rats with the expectation that high radioactivity accumulation would be observed in the regions where RIPK1 would be highly expressed. PET scans were performed 4 days after intracerebral administration of 50 μg lipopolysaccharide (LPS) on right striatum in rats. In both sides of striatum, radioactivity of [^11^C]GG502 peaked at 1 min (standardized uptake value (SUV) 3), followed by a rapid washout (Fig. 6). [^11^C]GG502 showed a good brain permeability, but no significant increased uptake was observed (only ~ 8% increase in averaged radioactivity from 30 to 120 min) in the LPS-treated side in the striatum. After reviewing these results, we decided not to pursue further blocking studies because it was thought that the uptake increases in LPS-treated side seen at baseline were too small in magnitude to show blocking effects if any.

LPS: lipopolysaccharide, SUV: standardized uptake value.

### Metabolite analysis of [^11^C]GG502 in rat plasma and brain

**Fig. 7:**
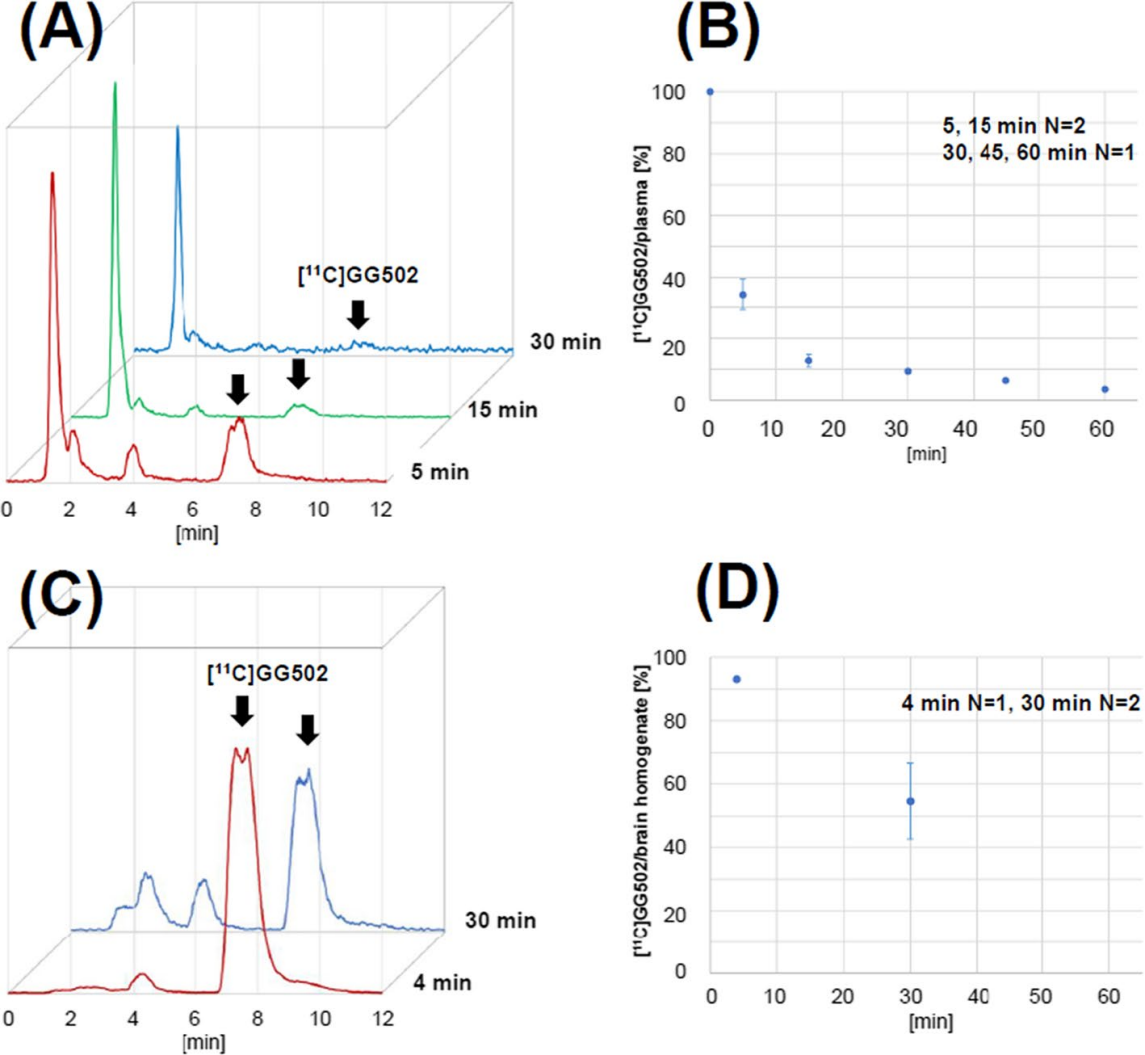
Radio-metabolite analysis in rat plasma and in rat brain homogenates. **A** Radio-HPLC charts of plasma samples taken at 5, 15, and 30 min after intravenous injection of [^11^C]GG502. **B** Time course of parent fraction in plasma. **C** Radio-HPLC charts of brain samples taken at 4 min and 30 min after intravenous injection of [^11^C]GG502. **D** Time course of parent fraction in the brain homogenates

We conducted metabolite analyses of [^11^C]GG502 in rat plasma and brain. In plasma, three metabolites were observed at earlier retention time points than those of the parent (Fig. 7A). At 30 min after administration of [^11^C]GG502, ~10% of radioactivity in plasma was from that of unmetabolized parent [^11^C]GG502 (Fig. 7B). In the brain, the same metabolites as in plasma except the one at 1.5 min retention time were observed (Fig. 7C). At 30 min after administration of [^11^C]GG502, ~55% of radioactivity was from unmetabolized [^11^C]GG502 (Fig. 7D).

### Discussion

In this study, we synthesized and evaluated a novel PET ligand aiming at autoradiography and imaging of RIPK1 in the brain by modification of GSK’963. We successfully [^11^C]methyl-labeled GSK’963 ([^11^C]GG502) with a high radiochemical purity. GG502 showed comparable RIPK1 inhibitory activity to its original compound, GSK’963. Brain PET imaging of [^11^C]GG502 showed good brain permeability, but [^11^C]GG502 did not show any sufficient amount of in vivo specific binding to RIPK1 in the brain, despite the fact that the autoradiography showed evidence of blocking effects for [^11^C]GG502, indicating the presence of specific binding. In vivo, [^11^C]GG502 was rapidly metabolized to give a significant amount of radiometabolites entering into the brain, which may be contributing to the fact that non-specific binding of [^11^C]GG502 to RIPK1 was detected in this study. Further efforts appear needed to develop RIPK1 radioligands with a higher binding affinity and preferably without radiolabeled metabolites entering into the brain.

We chose GSK’963 as a lead compound for our PET ligand with an expectation that it should have a good brain permeability for the following reason. The chemical structure of GSK’963 has the central nervous system PET multiparameter optimization (CNS PET MPO) score of 3.25. This score is considered to be a predictive index of brain permeability and it is calculated by the sum of six parameters (clogP, clogD, molecular weight, topological polar surface area, hydrogen bonding donor, and p*K*_a_) (Zhang et al. 2013). CNS PET MPO scores > 3 has been reported to increase the probability of success for CNS PET ligand development. The good brain permeability of [^11^C]GG502 demonstrated in monkey and rat PET imaging in this study reflects the calculated score (3.03) of GG502.

Autoradiography showed blocking effects in the mouse spleen and human brain with [^11^C]GG502, indicating the possibility of specific binding of [^11^C]GG502 to RIPK1 in vitro. However, in the autoradiography of human brain sections, radioactivity was not completely blocked (Fig. 4 right bottom). The residual radioactivity uniformly presented in the brain sections after blocking represents nonspecific binding of GG502. Although our PET ligand satisfied the first requirement of good brain penetration, it failed to meet the second critical requirement of the detectable presence of specific binding to RIPK1 in vivo. In the LPS-induced acute neuroinflammation model rats, no significant increased uptake was observed with [^11^C]GG502 in the striatum ipsilateral to the LPS injection. The reason for this finding may be related to the fact that the expression of RIPK1 in the striatum might not be high enough to be detected by the ligand, because the density of RIPK1 in mouse brain and human brain is known to be very low (1.54 parts per million [ppm] and 7.47 ppm that was equivalent to 20 and 98 fmol/mg of brain protein, respectively) relative to the binding affinity of our PET ligand (Wang et al. 2015; Huang et al. 2023).

Binding affinity and selectivity of GG502 for RIPK1 have not yet been measured. However, GG502 and GSK’963 should have a comparable binding affinity and selectivity for RIPK1 because GG502 showed a similar inhibitory activity against RIPK1 to that of GSK’963 (*IC*_50_: 44 nM for GG502 and 52 nM for GSK’963). If the binding affinity of GG502 was similar to that of GSK’963 (29 nM), the *B*_max_/*K*_D_ value was calculated to be ~ 0.7 for mouse brains and ~ 3.3 for human brains. Those *B*_max_/*K*_D_ values may not be high enough for a feasible PET ligand to be detected in in vivo PET imaging.

In the monkey brain, [^11^C]GG502 PET showed increased uptake after pre-administration of GG502. This increase could be explained by increased radioactivity by increased radioligand availability that exceeded the possibly small decrease of radioactivity by the blocking of [^11^C]GG502 specific binding. The increased radioligand availability can be caused by increased free fraction of [^11^C]GG502, peripheral blocking, and inhibition of a brain efflux transporter by the blocking agent (Altomonte et al. 2023; Kawamura et al. 2010).

The failure to detect specific binding in vivo in the brain could also be explained by the radiometabolites accumulation in the brain. We found rapid metabolism of [^11^C]GG502 in the rat plasma, and 50% of radioactivity was from radiometabolites in the rat brain at 30 min after the injection. Those radiometabolites were likely ^11^C-hydroxymethyl and ^11^C-carboxyl compounds in which the *meta*-methyl group of [^11^C]GG502 was oxidated based on the previous report of radiometabolism of ^11^C-methyl group of [^11^C]PE2I (Shetty et al. 2007).

In general, for successful PET imaging of the target molecules such as RIPK1 with reversibly binding radioligands, several requirements must be met, which include a sufficiently high specific binding affinity for the target (low *IC*_50_), low non-specific binding activity, no radio-metabolites entering the brain, good brain permeability and sufficiently high densities of the target molecule in the brain. In this PET study, the specific binding component of PET signals was not definitely detectable, i.e., very small part of the entire PET signals despite [^11^C]GG502’s high affinity for RIPK1 and its good brain permeability due to the fact that this ligand did not satisfy the rest of the requirements, high nonspecific binding, radiometabolite activity and overall very low densities of RIPK1 in the brain. Probably, of these factors, radiometabolite activity in the brain was the major contributor to mask the specific binding signal detection, because specific binding was indeed detectable in our autoradiography.

### Conclusions

We synthesized and evaluated a novel PET ligand based on the slight modification of the GSK’963 structure for imaging of RIPK1 in the brain. Although in autoradiography [^11^C]GG502 showed evidence of specific binding, actual PET imaging failed to detect any evidence of specific binding to RIPK1 despite its good brain permeability. [^11^C]GG502 was rapidly metabolized to give a significant amount of radiometabolites entering into the brain, which significantly contributes to the overall PET signal, making the relative proportion of specific binding signal very small. Further development of radioligands with a higher binding affinity for RIPK1 in vivo and more stable metabolism than the current compound would be required.

## Methods

### General remarks

Unless otherwise noted, starting materials and reagents purchased from Sigma-Aldrich Japan (Tokyo, Japan), FUJIFILM Wako Pure Chemical Corporation (Osaka, Japan), Tokyo Chemical Industry (Tokyo, Japan), KANTO CHEMICAL (Tokyo, Japan), and NACALAI TESQUE (Kyoto, Japan) were purchased commercial sources and used without further purification. Anhydrous solvents were obtained from FUJIFILM Wako Pure Chemical Corporation. Silica gel chromatography was performed using standard techniques. ^1^H and ^13^C nuclear magnetic resonance (NMR) spectra were recorded on a JEOL α-400 spectrometer. The chemical shifts are expressed in ppm downfield from tetramethylsilane (δ 0.00 and δ 0.00) or in ppm relative to CDCl_3_ (δ 7.26 and δ 77.0) in ^1^H and ^13^C NMR, respectively. The abbreviations s, d, t, q, and m signify singlet, doublet, triplet, quartet, and multiplet, respectively. High-resolution mass spectra were measured on a JEOL JMS-700/GI. Radioactivity was quantified with a dose calibrator (ATOMLAB 500, Biodex Medical Systems, Inc., NY, USA). The analytical radio-HPLC system used for the ^11^C-labeled product consisted of a radioactivity HPLC flow monitor with a 2″ × 2″ NaI scintillation detector (Gabi Star, Elysia-raytest GmbH, Straubenhardt, Germany) and an HPLC system (LC-20, Shimadzu, Kyoto, Japan). Optical rotations were measured on a JASCO P-2300 polarimeter equipped with 589 nm laser. Measurements were performed at 20 °C, using chloroform as solvent and reference. The specific rotation was calculated from the measured optical rotation α as follows: [α]_D_^20^ = 100*α*/(*l*•*c*) with specific rotation, *l* path length and *c* concentration. The clogP of GG502 was calculated based on its chemical structure by ChemDraw 20.1.1 software.

### Separation of (*S*)- and (*R*)-2,2-dimethyl-1-(5-(3-bromophenyl)-4,5-dihydro-1*H*-pyrazol-1-yl)propan-1-one (5a and 5b)

2,2-Dimethyl-1-(5-(3-bromophenyl)-4,5-dihydro-1*H*-pyrazol-1-yl)propan-1-one (3.23 g, 10.4 mmol) was separated into its enantiomers via chiral chromatography preparative HPLC under the following conditions: preparative HPLC column: CHIRAL ART Cellulose-SC (5 μm, 30 mm internal diameter (i.d.) × 250 mm, YMC CO., LTD., Kyoto Japan); flow rate: 21.3 mL/min; eluent: *n*-hexane/2-propanol = 70:30. Obtained were (*S*)-2,2-dimethyl-1-(5-(3-bromophenyl)-4,5-dihydro-1*H*-pyrazol-1-yl)propan-1-one (1.49 g, 46.1% yield), [α]_D_ = –123° (conc = 2.92 × 10^–3^, chloroform), ee = 99.9%, ^1^H NMR and (*R*)-2,2-dimethyl-1-(5-(3-bromophenyl)-4,5-dihydro-1*H*-pyrazol-1-yl)propan-1-one (1.45 g, 44.9% yield), [α]_D_ = 122° (conc = 2.52 × 10^–3^, chloroform), ee = 99.9%. The absolute configurations of the enantiomer were determined by optical rotation of GSK’963 [α]_D_ = –176° (conc = 2.74 × 10^–3^, chloroform) and GSK’962 [α]_D_ = 140° (conc = 3.65 × 10^–3^, chloroform).

### Radiosynthesis of (***S***)-2,2-dimethyl-1-(5-(3-([^11^C]methyl)phenyl)-4,5-dihydro-1***H***-pyrazol-1-yl)propan-1-one ([^11^C]GG502)

The reaction mixture of tris(dibenzylideneacetone)dipalladium (3.8 mg, 4.1 μmol), tris(*o*-toly)phosphine (3.9 mg, 13 μmol), potassium carbonate (1.0 mg, 7.2 μmol) and the corresponding pinacol boronate precursor **6a** (0.4 mg, 1.1 μmol) in 0.42 mL of *N*,*N*-dimethylformamide/water (6:1 *v*/*v*) was set in a second vial and kept below − 10 °C while waiting for the [^11^C]iodomethane preparation. [^11^C]iodomethane was distilled into the second vial under nitrogen gas flow. The resulting mixture was then quickly heated to 60 °C and kept for 4 min with 2-s mixing by 30 mL/min nitrogen flow before 3 min of the end of heating. After diluting by 1.0 mL of HPLC eluent, the radioactive mixture was applied to a semi-preparative HPLC column. HPLC purification was performed using a JASCO HPLC System (JASCO, Tokyo, Japan) under the following conditions: preparative HPLC column: CAPCELL PAK C18 UG 120 (5 μm, 10 mm i.d. × 250 mm, Osaka Soda CO., LTD., Osaka, Japan); eluent: acetonitrile/20 mM sodium dihydrogen phosphate = 55:45 (*v*/*v*); flow rate: 6 mL/min; detection: ultraviolet (UV), 254 nm; retention time: 10.5 min. The desired fraction was collected into a flask followed by evaporation and dissolve with 4 mL of saline containing of 0.5% (*v*/*v*) tween 80, and the product was sterilized by filtration over a 0.22-μm Millex GV filter (Merck Millipore Ltd., USA). The total synthesis time, including HPLC purification and radiopharmaceutical formulation for intravenous administration, was 29 min. The isolated radioactivity was 1.2 GBq at the end of synthesis and the molar activity was 52 GBq/μmol. The decay-corrected radiochemical yield was 6%, which was calculated on the basis of the radioactivity of [^11^C]carbon dioxide trapped in a first vial. The chemical identified as [^11^C]GG502 was confirmed by co-injection with non-radiolabeled GG502 on analytical HPLC (see Additional file 1: Fig. S6). The chemical purity analyzed at 254 nm and radiochemical purity were 89% and 97%, respectively. HPLC analysis was performed under the following conditions: Column: CAPCELL PAK C18 UG 120 (5 μm, 4.6 mm i.d. × 250 mm, Osaka Soda CO., LTD., Osaka, Japan); eluent: acetonitrile/20 mM sodium dihydrogen phosphate = 55:45 (*v*/*v*); flow rate: 1.5 mL/min; detection: UV, 254 nm; retention time, 8 min.

### Inhibitory activity assay for RIPK1

The inhibitory activity assay was performed using a commercially available RIPK1 kinase Assay Kit (#79,560, BPS Bioscience, USA) according to its protocol. 0.10 μg recombinant human RIPK1 protein was diluted in the reaction buffer (Kinase assay buffer, 10 mM dithiothreitol) and incubated with 20 μM adenosine triphosphate, RIPK1 substrate (myelin basic protein) and inhibitors (1.0 μM–0.1 nM in final concentration). After incubation for 45 min at 30 °C, the reaction was stopped by the addition of ADP-Glo® Reagent (V6930, Promega Corporation, WI, USA) and incubated for 45 min at room temperature under shading conditions. After the incubation, kinase detection reagent (V6930, Promega Corporation, WI, USA) was added to the reaction and incubated for 45 min at room temperature under shading. Luminescence was measured with a microplate reader (SpectraMax® Paradigm®, MOLECULAR DEVICES). Curve fitting was performed using GraphPad Prism® sigmoidal dose–response software (GraphPad Software, CA, USA).

### Animals

Crl:CD Sprague–Dawley (SD) rats (male, 9 weeks old, Charles River Laboratories Japan Inc., Yokohama, Japan), Fischer 344 (F344/NSlc) rats (male, 8–10 weeks old, Japan SLC Inc., Hamamatsu, Japan), C57/B6 mice (CLEA Japan Inc., Tokyo, Japan) and a rhesus monkey (3.6 kg, 4.7 years old) were used for the current study. The animals used here were maintained and handled in accordance with the National Research Council's Guide for the Care and Use of Laboratory Animals and our institutional guidelines. Protocols for the present animal experiments were approved by the Animal Ethics Committee of the National Center for Gelotology and Geriatrics for the rat experiments and the National Institutes for Quantum Science and Technology for the mouse and monkey experiments.

### Autoradiography

Healthy mouse spleens were removed rapidly and then frozen immediately in powdered dry ice. Postmortem human brains were obtained from autopsies carried out at the Center for Neurodegenerative Disease Research of the University of Pennsylvania Perelman School of Medicine on subjects without any significant brain pathologies Autoradiography was performed using fresh frozen sections with 20-μm-thick for mouse spleen and human brains. Sections were pre-incubated for 30 min and then incubated in fetal bovine serum containing [^11^C]GG502 at room temperature for 60 min without or with 10 µM GG502 as a blocking agent added in the serum. The samples were then rinsed with ice-cold serum twice for 2 min each, and dipped into ice-cold water for 10 s. The sections were subsequently dried by treating with warm air and were then exposed to an imaging plate (BAS-MS2025, Fuji Film, Tokyo, Japan). The imaging plate was scanned with a BAS-5000 system (Fuji Film, Tokyo, Japan) to acquire autoradiograms.

### Evaluation in vivo

#### PET brain imaging in a monkey

The monkey was initially anesthetized with an intramuscular injection of ketamine (5–10 mg/kg) and xylazine (0.2–0.5 mg/kg) and then intubated and kept anesthetized with 1–2% isoflurane. A 120-min dynamic PET acquisition using a microPET Focus 220 system (Siemens, Knoxville, TN) was started immediately after the bolus injection of [^11^C]GG502 (331 and 347 MBq corresponding to 3.8 and 4.3 nmol, respectively) without or with pre-administration of a blocking agent. A transmission scan with a ^68^Ge-^68^ Ga point source was carried out before PET acquisitions to generate attenuation map. In the blocking study, 1.0 mg/kg GG502 was injected intravenously at 5 min prior to the PET acquisitions.

#### Rat model of acute neuroinflammation

Two SD rats (male, 9 weeks old, 296 and 303 g) were stereotaxically injected with lipopolysaccharide from *Escherichia coli* (O55:B5) (0.05 mg in 4 µL saline, #L2637, Merck, Darmstadt, Germany) in the right striatum.

#### PET brain imaging in rats

One of the two above mentioned inflammation model rats with apparent neuroinflammation confirmed with [^11^C]DPA-713 imaging (see Additional file 1: Fig. S9) was used for brain imaging at 4 days after the LPS administration. Rat was scanned for 120 min on a small animal PET scanner (FX3200, TriFoil Imaging, CA, USA) after an injection of [^11^C]GG502 (33.4 MBq, 0.7 nmol) via the tail vein under the isoflurane anesthesia (~ 2.0%). All PET images were reconstructed with the three-dimensional ordered subset expectation maximization method (4 subsets and 20 iterations; voxel size: 0.6 × 0.5 × 0.5 mm with the resolution of 0.92 mm full width at half maximum at the center of view).

#### Metabolite analysis of [^11^C]GG502 in rat blood and brain

Blood metabolite analyses were performed on two F344/NSlc rats (male, 8 weeks old, 146–149 g). Two rats were intravenously injected via the tail vein with [^11^C]GG502 (94.2 and 223 MBq, 2.3 and 4.1 nmol) under the isoflurane anesthesia (~ 2.0%). Arterial blood was sampled at five time points (5, 15, 30, 45 and 60 min after the injection). The blood samples were centrifuged at 12,000 revolutions per minute (rpm) for 3 min at 4 °C to separate out the plasma. The supernatant (0.10 mL) was resuspended in acetonitrile (0.15 mL), and the mixture was placed on ice for 3 min after inverted mixing and deproteinized by centrifugation at 12,000 rpm for 3 min at 4 °C.

Brain metabolite analyses were performed on two healthy F344/NSlc rats (male, 9 and 10 weeks old, 192 and 207 g). Two rats were intravenously injected via the tail vein with [^11^C]GG502 (142 and 304 MBq, 3.1 and 6.4 nmol) under the isoflurane anesthesia (~ 2.0%) and sacrificed by decapitation at 4 and 30 min after the injection. A half-brain hemisphere including cerebellum was homogenized in radio-immunoprecipitation assay (RIPA) buffer (4.0 mL, Fujifilm Wako Pure Chemical Corporation, Osaka, Japan) on ice. The homogenate was centrifuged at 12,000 rpm for 3 min at 4 °C, and the supernatant was collected, resuspended in acetonitrile (4.0 mL) on ice, and deproteinized by centrifugation at 12,000 rpm for 3 min at 4 °C.

For both blood and brain metabolite analyses, the supernatant obtained from the plasma and brain homogenate was injected into a radio-HPLC (Prominence LC-20 system, Shimadzu, Kyoto, Japan and FC-4100, Eckert & Ziegler Radiopharma, Berlin, Germany) equipped with a Cadenza CD-C18 column (3 μm, 10 mm i.d. × 75 mm, Imtakt Corporation, Kyoto, Japan) and eluted by acetonitrile/water = 60:40 (*v*/*v*). The flow rate was 3.0 mL/min. The unmetabolized fraction was calculated as ratios of the decay-corrected peak area between the unmetabolized [^11^C]GG502 peak and the total peaks. Time courses of unmetabolized plasma [^11^C]GG502 fraction were averaged.

### Supplementary Information

**Additional file 1:** Fig. S1-S2 and S3-S4 for NMR spectra of **6a** and non-radioactive GG502, respectively.

## Data Availability

The datasets generated and/or analyzed during the current study are available from the corresponding author on reasonable request.

## Abbreviations

AD: Alzheimer's disease
*B*_max_: Available receptor site concentration
CNS: Central nervous system
*EC*_50_: 50% Effective concentration
HPLC: High performance liquid chromatography
*IC*_50_: 50% Inhibitory concentration
i.d.: Internal diameter
*K*_D_: Equilibrium dissociation constant
LPS: Lipopolysaccharide
MPO: Multiparameter optimization
NMR: Nuclear magnetic resonance
PET: Positron emission tomography
ppm: Parts per million
RIPK1: Receptor interacting protein kinase 1
rpm: Revolutions per minute
SUV: Standardized uptake value
UV: Ultraviolet
*v*/*v*: Volume per volume
%ID/cc: Percent injected dose per unit volume
