# Supplementary material for: Synthesis and evaluation of a novel PET ligand, a GSK’963 analog, aiming at autoradiography and imaging of the receptor interacting protein kinase 1 in the brain
Source: EJNMMI Radiopharm Chem. 2023 Oct 18;8:31. doi: 10.1186/s41181-023-00217-z (PMC10584749; doi:10.1186/s41181-023-00217-z)
Supplement: Supplementary file 1 — Additional file 1: Fig. S1-S2 and S3-S4 for NMR spectra of 6a and non-radioactive GG502, respectively. [file 41181_2023_217_MOESM1_ESM.docx]

Supplementary information

Synthesis and evaluation of a novel PET ligand, a GSK’963 analog, aiming at autoradiography and imaging of the receptor interacting protein kinase 1 in the brain.

Hiroshi Ikenuma^1^, Aya Ogata^1,2^, Hiroko Koyama^3^, Bin Ji^4,5^, Hideki Ishii^6^, Takashi Yamada^1^, Junichiro Abe^1^, Chie Seki^4^, Yuji Nagai^4^, Masanori Ichise^1^, Takafumi Minamimoto^4^, Makoto Higuchi^4^, Ming-Rong Zhang^6^, Takashi Kato^1^, Kengo Ito^1^, Masaaki Suzuki^1^, Yasuyuki Kimura*^1^

1. Department of Clinical and Experimental Neuroimaging, Center for Development of Advanced Medicine for Dementia, National Center for Geriatrics and Gerontology (NCGG), Obu, Japan
2. Department of Pharmacy, Faculty of Pharmacy, Gifu University of Medical Science (GUMS), Kani, Japan
3. Department of Chemistry and Biomolecular Science, Faculty of Engineering, Gifu University, Gifu, Japan
4. Department of Functional Brain Imaging, National Institutes for Quantum Science and Technology (QST), Chiba, Japan
5. Department of Radiopharmacy and Molecular Imaging, School of Pharmacy, Fudan University, Shanghai, China
6. Department of Advanced Nuclear Medicine Sciences, National Institutes for Quantum Science and Technology (QST), Chiba, Japan

**First Author:**Hiroshi Ikenuma

**Address correspondence and reprint requests:**
Yasuyuki Kimura, MD, PhD
Department of Clinical and Experimental Neuroimaging, Center for Development of Advanced Medicine for Dementia, National Center for Geriatrics and Gerontology

7-430 Morioka-cho, Obu, Aichi 474-8511, Japan

TEL: +81562462311, FAX: +81562446596

E-mail: [yazkim@ncgg.go.jp](mailto:yazkim@ncgg.go.jp)

**Table of Contents**

| Topic | Display item(s) | Page |
| --- | --- | --- |
| Chemistry | Figure S1, S2, S3, S4 | S3 |
| Representative semi-preparative HPLC chromatogram of the [^11^C]GG502 fraction | Figure S5 | S7 |
| Representative analytical HPLC chromatogram of the formulated [^11^C]GG502 | Figure S6 | S8 |
| Calibration curve for the molar activity of [^11^C]GG502 | Figure S7 | S9 |
| Autoradiography of [^11^C]GG502 in mouse spleen with adjusted contrast | Figure S8 | S10 |
| PET imaging of [^11^C]DPA-713 in rat brain 4 days after lipopolysaccharide administration | Figure S9 | S11 |

**Chemistry**

**(*S*)-2,2-dimethyl-1-(5-(3-(4,4,5,5-tetramethyl-1,3,2-dioxaborolan-2-yl)phenyl)-4,5-dihydro-1*H*-pyrazol-1-yl)propan-1-one (6a)**

^1^H NMR (400 MHz, CDCl_3_): δ = 1.32 (s, 6H, CH_3_), 1.33 (s, 6H, CH_3_), 1.34 (s, 9H, CH_3_), 2.65 (ddd, *J* = 18.4, 4.4, 1.6 Hz, 1H, CH_2_), 3.28 (ddd, *J* = 18.8, 12.0, 1.6 Hz, 1H, CH_2_), 5.40 (dd, *J* = 11.8, 4.4 Hz, 1H, CH), 6.88–6.90 (m, 1H, CH=N), 7.16–7.19 (m, 1H, aromatic), 7.28–7.32 (m, 1H, aromatic), 7.62–7.68 (m, 2H, aromatic); ^13^C NMR (100 MHz, CDCl_3_): d = 24.89 (4C), 26.96 (3C), 39.80, 42.33, 59.15, 77.21 (2C), 83.77, 127.55, 128.32, 131.83, 133.83, 141.89, 144.20, 175.65; HR-MS (EI+, 100% acetone): *m*/*z*: calcd for C_20_H_29_BN_2_O_3_ 356.2271 found 356.2311.

**Figure S1.** ^1^H NMR spectrum of **6a**.

**Figure S2.** ^13^C NMR spectrum of **6a**.

***(S)-*2,2-dimethyl-1-(5-(*m*-tolyl)-4,5-dihydro-1*H*-pyrazol-1-yl)propan-1-one (GG502)**

^1^H NMR (400 MHz, CDCl_3_): δ = 1.33 (s, 9H, CH_3_), 2.31 (s, 3H, CH_3_), 2.65 (ddd, *J* = 18.6, 5.0, 1.8 Hz, 1H, CH_2_), 3.28 (ddd, *J* = 18.6, 12.2, 1.8 Hz, 1H, CH_2_), 5.34 (dd, *J* = 12.0, 4.8 Hz, 1H, CH), 6.89–6.91 (m, 3H, CH=N and aromatic), 7.03–7.05 (m, 1H, aromatic), 7.17–7.21 (m, 1H, aromatic); ^13^C NMR (100 MHz, CDCl_3_): δ = 21.49, 26.97 (3C), 39.79, 42.38, 59.13, 121.97, 125.89, 128.16, 128.81, 138.41, 142.64, 144.23, 175.64; HR-MS (EI+, 100% acetone): *m*/*z*: calcd for C_15_H_20_N_2_O 244.1576 found 244.1578.

**Figure S3.** ^1^H NMR spectrum of **GG502**.

**Figure S4.** ^13^C NMR spectrum of **GG502**.

**Figure S5.** Representative semi-preparative HPLC chromatogram of the [^11^C]GG502 fraction. The semi-preparative HPLC was performed under the following conditions: preparative HPLC column: CAPCELL PAK C18 UG 120 (5 μm, 10 mm i.d. × 250 mm, Osaka Soda CO., LTD., Osaka, Japan); eluent: acetonitrile/20 mM sodium dihydrogen phosphate = 55:45 (*v*/*v*); flow rate: 6 mL/min; detection: ultraviolet (UV), 254 nm; retention time: 10.5 min.

**Figure S6.** Representative analytical HPLC chromatogram of the formulated [^11^C]GG502 (UV(A) and radioactivity (B)) and the formulated [^11^C]GG502 with nonradioactive GG502 (UV (C) and radioactivity (D)). The analytical HPLC was performed under the following conditions: preparative HPLC column: CAPCELL PAK C18 UG 120 (5 μm, 4.6 mm i.d. × 250 mm, Osaka Soda CO., LTD., Osaka, Japan); eluent: acetonitrile/20 mM sodium dihydrogen phosphate = 55:45 (*v*/*v*); flow rate: 1.5 mL/min; detection: ultraviolet (UV), 254 nm; retention time: 8 min.

**Figure S7.** HPLC calibration curve to determine amount of GG502 used in the calculation of molar activity of [^11^C]GG502 in the range of 0.02-1.52 nmol. HPLC analysis was performed under the following conditions: Column: CAPCELL PAK C18 UG 120 (5 μm, 4.6 mm i.d. × 250 mm, Osaka Soda CO., LTD., Osaka, Japan); eluent: acetonitrile/20 mM sodium dihydrogen phosphate = 55:45 (*v*/*v*); flow rate: 1.5 mL/min; detection: UV, 254 nm; retention time, 8 min.

**Figure S8.** Autoradiography of [^11^C]GG502 in mouse spleen without (left) and with addition of non-radioactive GG502 (10 μM) as a blocking agent (right) with adjusted contrast.

**Figure S9.** PET imaging of [^11^C]DPA-713 in rat brain 4 days after lipopolysaccharide administration. Coronal section PET images (averaged from 0 to 20 min) displayed with color scale units in standardized uptake values overlaid on template MR images.
